# Supplementary material for: Characterization and Electrical Properties of PVA Films with Self-Assembled Chitosan-AuNPs/SWCNT-COOH Nanostructures
Source: Materials (Basel). 2020 Sep 17;13(18):4138. doi: 10.3390/ma13184138 (PMC7560243; doi:10.3390/ma13184138)
Supplement: Supplementary file 1 [file materials-13-04138-s001.pdf]

materials

Supplementary Materials

Characterization and Electrical Properties of PVA Films with Self-Assembled Chitosan-AuNPs/SWCNT-COOH Nanostructures

Israel Ceja [1], Karla Josefina González-Íñiguez [2], Alejandra Carreón-Álvarez [3], Gabriel Landazuri [4], Arturo Barrera [5], José Eduardo Casillas [6], Víctor Vladimir A. Fernández-Escamilla [6] and Jacobo Aguilar [6,*]

[1] Departamento de Física, Centro Universitario de Ciencias Exactas e Ingeniería, Universidad de Guadalajara, Blvd. M. García Barragán # 1421, C.P. 44430, Guadalajara, Mexico; iscean12@yahoo.com.mx

[2] Departamento de Química, Centro Universitario de Ciencias Exactas e Ingeniería, Universidad de Guadalajara, Blvd. M. García Barragán # 1421, C.P. 44430, Guadalajara, Mexico; karlajgi@hotmail.com

[3] Departamento de Ciencias Naturales y Exactas, Centro Universitario de los Valles, Universidad de Guadalajara, Carretera Guadalajara-Ameca Km. 45.5, C.P. 46600, Ameca, Mexico; ale_carreon_a@yahoo.com.mx

[4] Departamento de Ingeniería Química, Centro Universitario de Ciencias Exactas e Ingeniería, Universidad de Guadalajara, Blvd. M. García Barragán # 1421, C.P. 44430, Guadalajara, Mexico; gabriel.landazuri@academicos.udg.mx

[5] Departamento de Ciencias Básicas, Centro Universitario de la Ciénega, Universidad de Guadalajara, Avenida Universidad No. 1115, C.P. 47810, Ocotlán, Mexico; arturobr2003@yahoo.com.mx

[6] Departamento de Ciencias Tecnológicas, Centro Universitario de la Ciénega, Universidad de Guadalajara, Avenida Universidad No. 1115, C.P. 47810, Ocotlán, Mexico; duart_casillas@hotmail.com; vladkrm@hotmail.com; jax781023@hotmail.com

* Correspondence: jax781023@hotmail.com

Table S1. Equations for the calculate of *Dn*, *Dz* and *PDI*, from distributions of particle size by Dynamic Light Scattering (DLS).

Name	Equation
Number average particle size:	$Dn = \frac{\sum n_i Di}{\sum n_i}$
Weight average particle size:	$Dw = \frac{\sum n_i {D_i}^4}{\sum n_i D_i^3}$
z-Average particle size:	$Dz = \frac{\sum n_i {D_i}^6}{\sum n_i D_i^5}$
Polidispersity index:	$PDI = \frac{D_w}{D_n}$

The chitosan-AuNPs nanostructures, it was not possible to be identified by TEM, because the chitosan chains were separated from the AuNPs when was applied the electron beam accelerating voltage in order to increase the magnification, then temperature increased at the irradiated point and the chitosan chain were separated by electron beam, which through of the sample. Figure S1 shows the chitosan separated from the AuNPs and the agglomerated on the cover grid used in TEM.

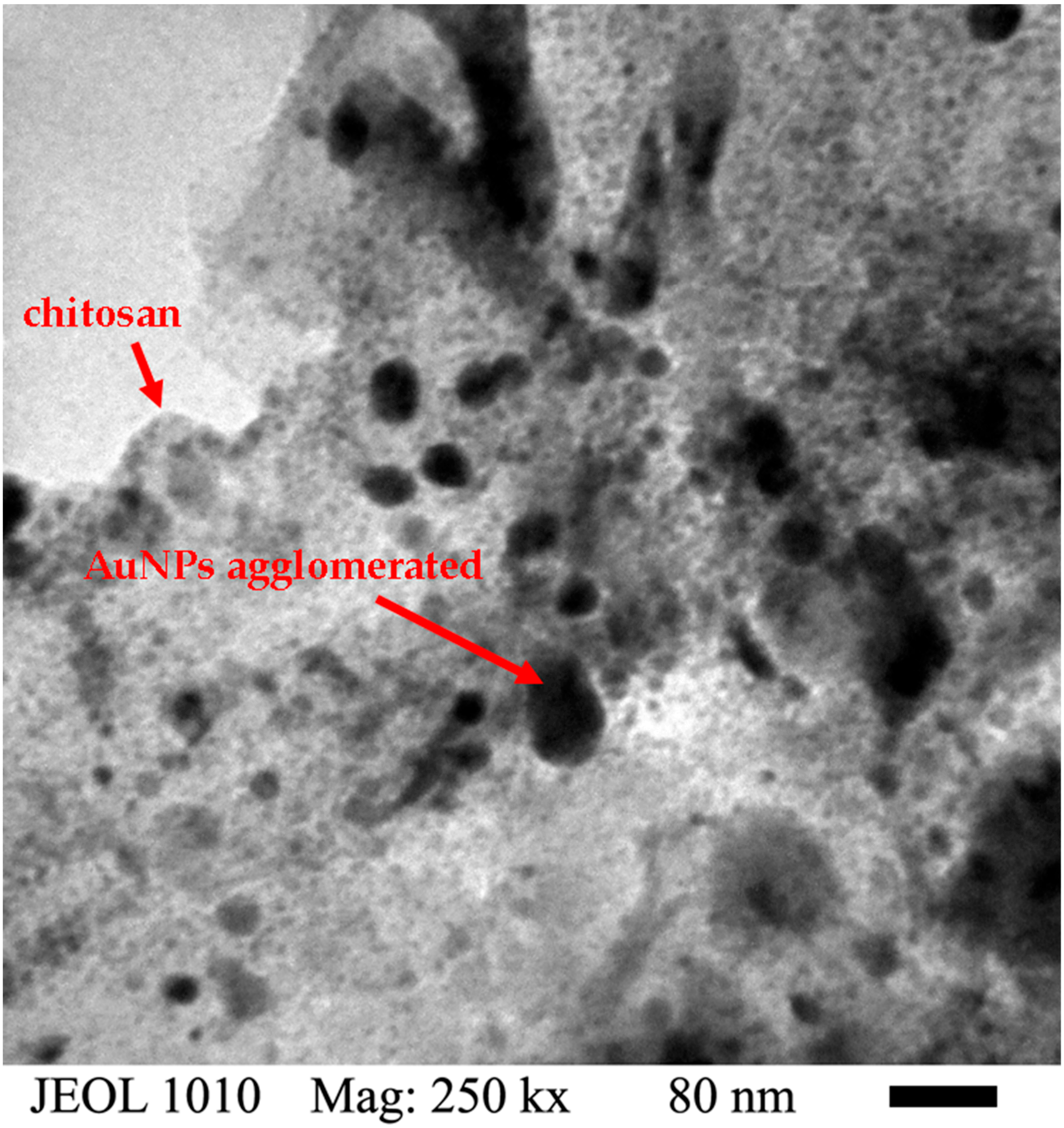

Figure S1. Photographs obtained by TEM of chitosan-AuNPs nanostructures collapsed on the copper grid for the concentration of 0.4mM $HAuCl_4{\cdot}3H_2O$.

Figures S2–S5 show the *Eg* calculations of the of PVA films with self-assembled chitosan-AuNPs/SWCNT-COOH nanostructures at the different $HAuCl_4{\cdot}3H_2O$ and SWCNT-COOH concentrations according to the Tauc's graph.

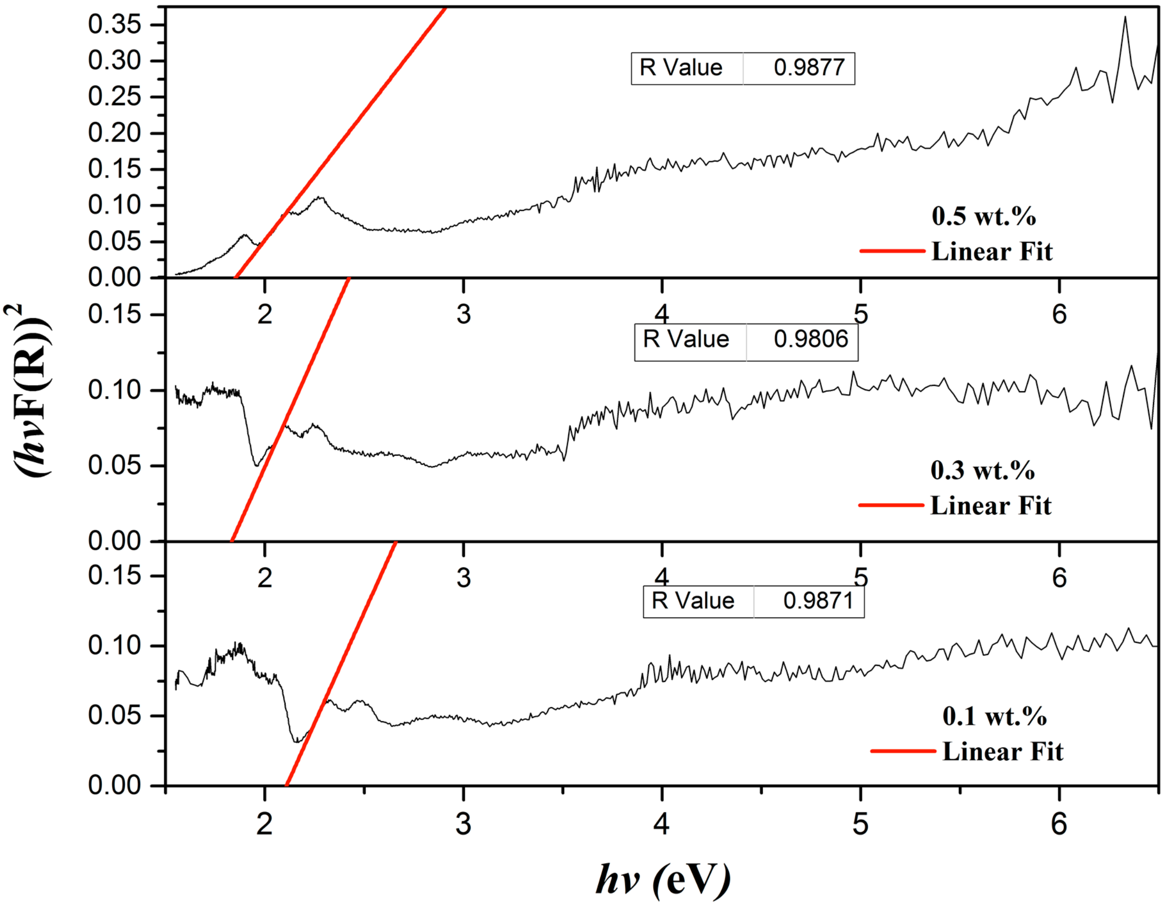

Figure S2. Eg calculations of 0.4 mM $HAuCl_4 \cdot 3H_2O$ concentration, and different SWCNT-COOH concentrations: 0.1, 0.3 and, 0.5 wt.%.

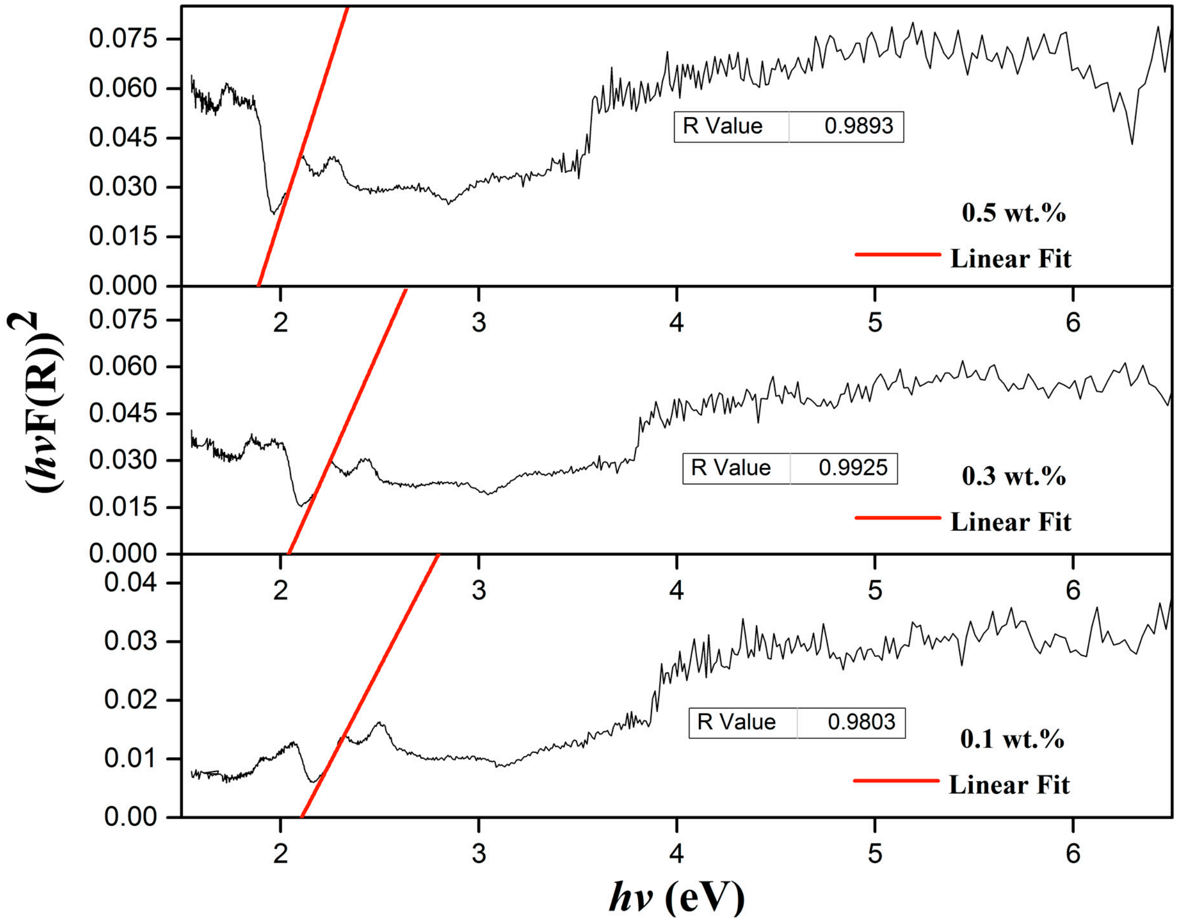

Figure S3. Eg calculations of 0.7 mM $HAuCl_4 \cdot 3H_2O$ concentration, and different SWCNT-COOH concentrations: 0.1, 0.3 and, 0.5 wt.%.

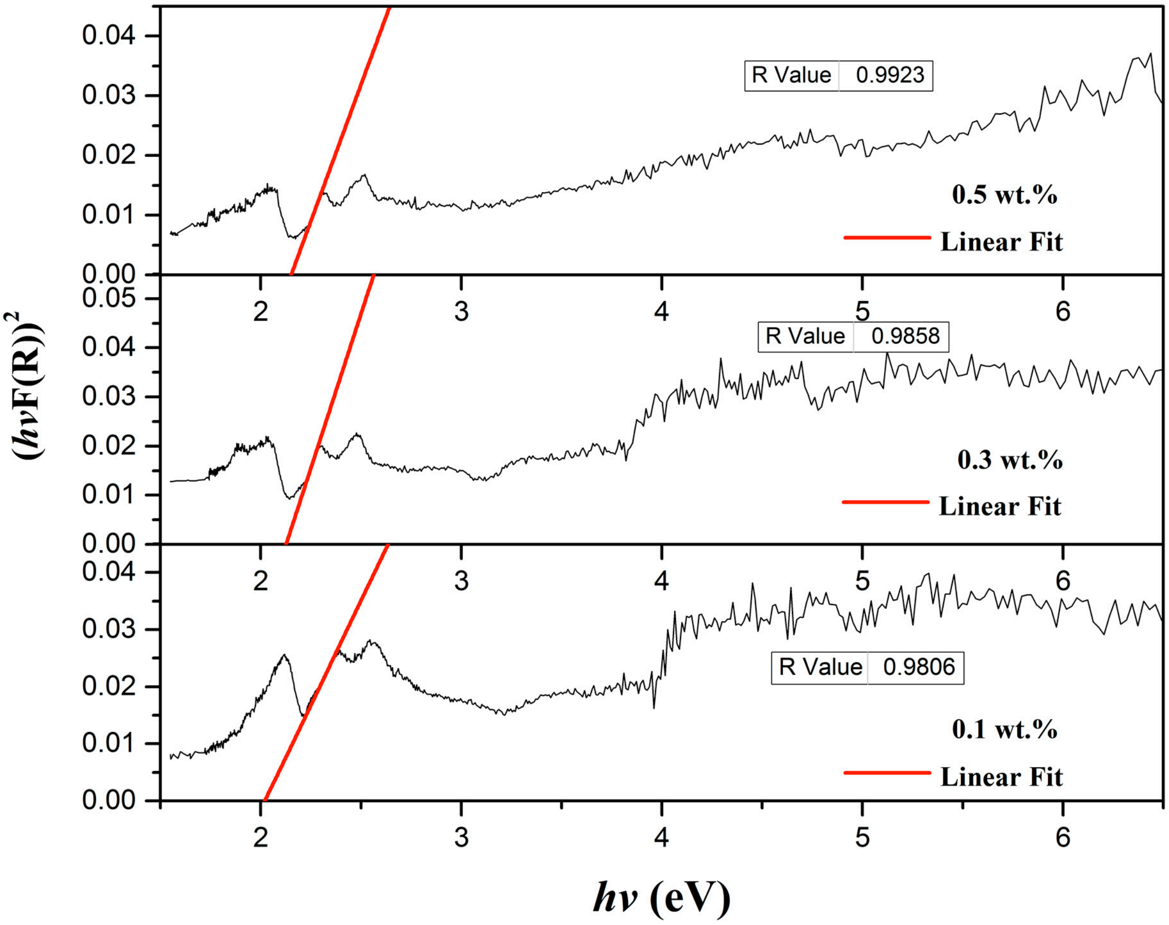

Figure S4. Eg calculations of 1.0 mM $HAuCl_4{\cdot}3H_2O$ concentration, and different SWCNT-COOH concentrations: 0.1, 0.3 and, 0.5 wt.%.

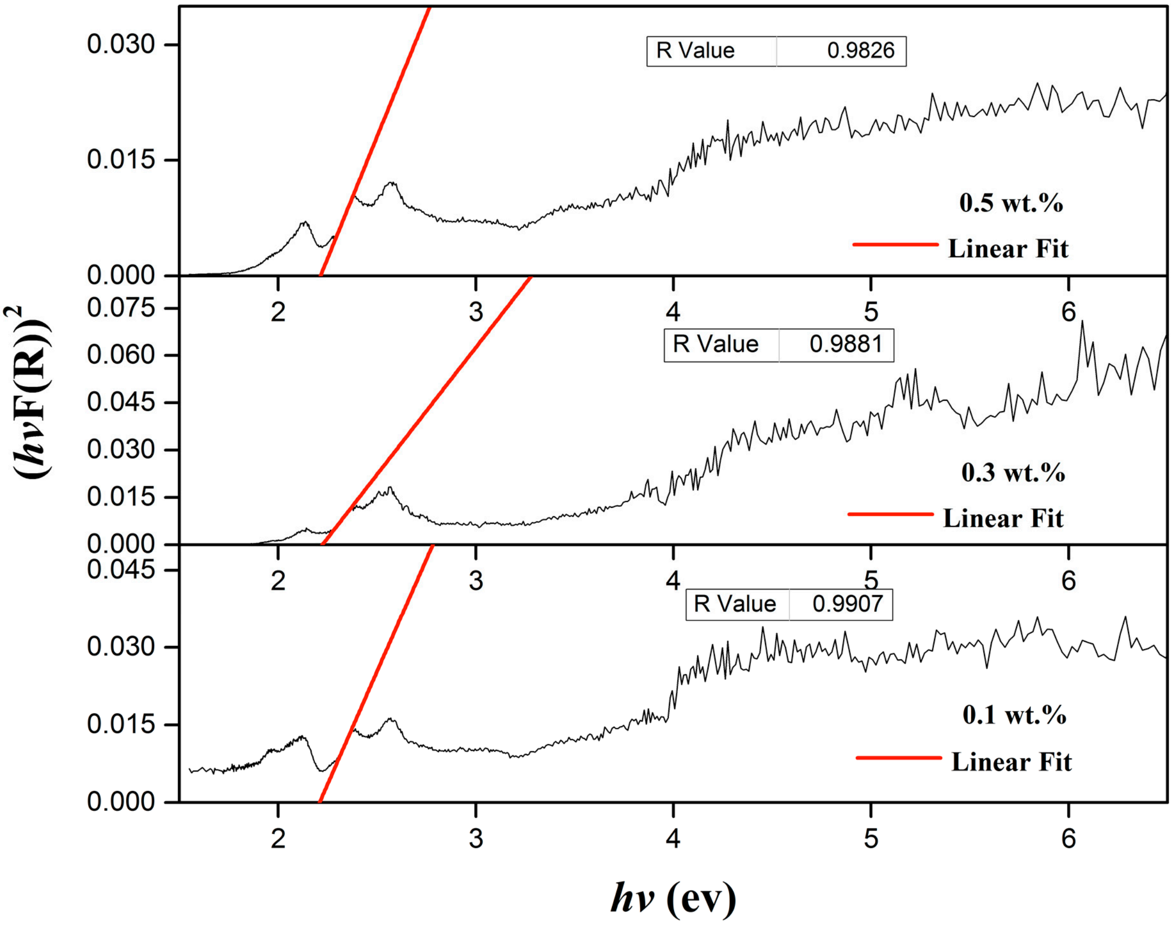

Figures S5. Eg calculations of 1.3 mM $HAuCl_4{\cdot}3H_2O$ concentration, and different SWCNT-COOH concentrations: 0.1, 0.3 and, 0.5 wt.%.
